# Prevalence of HAV and HEV in the patients presenting with acute viral hepatitis

**DOI:** 10.1186/1471-2334-12-S1-P30

**Published:** 2012-05-04

**Authors:** Pooja Rao, M Suchitra Shenoy, Shrikala Baliga, Annu Joon

**Affiliations:** 1 Department of Microbiology, Kasturba Medical College, Mangalore-575001, Karnataka, India

## Background

Hepatitis A and E are both enterically transmitted resulting in sporadic and epidemic forms of acute hepatitis in developing countries including India. This study was done to determine the prevalence of HAV and HEV in the patients presenting with acute viral hepatitis and also to determine co-infection of HAV and HEV in these patients.

## Methods

The cross-sectional study was conducted in Kasturba Medical College, Manglore. A non-random sampling of 958 patients presenting with acute viral hepatitis were included. On the basis of history, serum samples were analyzed for IgM anti HAV and IgM anti HEV for the detection of hepatitis A (HAV) and hepatitis E (HEV) respectively using commercially available ELISA kits.

## Results

The seroprevalence of HAV and HEV positive patients were 19.31% and 10.54% respectively. The prevalence of both HAV and HEV in patients with acute viral hepatitis was 11.5%. The prevalence of HAV and HEV among males (68% & 31%) was higher than in females (31% & 20%). HAV and HEV were predominantly seen among young adults. These infections were predominantly seen during later part of rainy and beginning of winter season.

## Conclusion

Though the prevalence of HAV is much higher than that of HEV, co infection rate of 11.5% mandates the screening for HEV which will be of immense importance in pregnant women and improving levels of personal hygiene among higher socio-economic population. These data will be essential for planning of future vaccination strategies and for better sanitation programme in this part of the country.

